# Task-Oriented Circuit Training as an Alternative to Ergometer-Type Aerobic Exercise Training after Stroke

**DOI:** 10.3390/jcm10112423

**Published:** 2021-05-30

**Authors:** Liam P. Kelly, Augustine J. Devasahayam, Arthur R. Chaves, Marie E. Curtis, Edward W. Randell, Jason McCarthy, Fabien A. Basset, Michelle Ploughman

**Affiliations:** 1 Recovery and Performance Laboratory, L.A. Miller Centre, Faculty of Medicine, Memorial University of Newfoundland, St. John’s, NL A1K 5A1, Canada; augustine.joshua@mun.ca (A.J.D.); ardac2@mun.ca (A.R.C.); marie.curtis@mun.ca (M.E.C.); jason.mccarthy@med.mun.ca (J.M.); 2 School of Human Kinetics and Recreation, Memorial University of Newfoundland, St. John’s, NL A1C 5S7, Canada; fbasset@mun.ca; 3 Discipline of Laboratory Medicine, Faculty of Medicine, Memorial University of Newfoundland, St. John’s, NL A1B 3V6, Canada; Ed.Randell@easternhealth.ca

**Keywords:** stroke, rehabilitation, aerobic exercise, secondary prevention, maximum oxygen uptake, task-oriented, circuit training

## Abstract

Moderate-intensity aerobic exercise training is an important treatment strategy to enhance functional recovery and decrease cardiometabolic risk factors after stroke. However, stroke related impairments limit access to ergometer-type exercise. The aims of the current study were (1) to evaluate whether our task-oriented circuit training protocol (intermittent functional training; IFT) could be used to sustain moderate-intensity aerobic workloads over a 10-week intervention period, and (2) to investigate its preliminary effects on cardiorespiratory fitness and metabolic profiles compared to constant-load ergometer-type exercise (CET). Forty chronic hemiparetic stroke survivors were randomized to receive 30 sessions of IFT or CET over ten weeks. Similar proportions of participants were randomized to IFT (7/19) and CET (9/18) sustained workloads associated with moderate-intensity aerobic exercise over the study period (*p* = 0.515). However, CET was associated with more substantial changes in maximal oxygen uptake (MD = 2.79 mL min^−1^ kg^−1^ CI: 0.84 to 4.74) compared to IFT (MD = 0.62 mL min^−1^ kg^−1^ CI: −0.38 to 1.62). Pre to post changes in C-reactive protein (−0.9 mg/L; *p* =0.017), short-term glycemia (+14.7 µmol/L; *p* = 0.026), and resting whole-body carbohydrate oxidation (+24.2 mg min^−1^; *p* = 0.046) were observed when considering both groups together. Accordingly, IFT can replicate the aerobic intensities sustained during traditional ergometer-type exercise training. More work is needed to evaluate the dose–response effects of such task-oriented circuit training protocols on secondary prevention targets across the continuum of stroke recovery.

## 1. Introduction

Stroke rehabilitative efforts are primarily directed toward the recovery of lost functions. Hence, best practice guidelines include 15 h of direct task-oriented therapy each week during inpatient/outpatient rehabilitation services [1]. Such treatment involves practicing relevant tasks, including moving from lying to sitting, stepping, and walking. As task-oriented activities delivered during neurorehabilitation programs are focused on restoring more normal movement patterns after stroke, they provide insufficient cardiovascular stress levels [2]. Accordingly, practitioners are recommended to incorporate an additional 150 min of moderate-intensity aerobic exercise each week during formalized care and encourage stroke survivors to remain active throughout the continuum of recovery [3,4]. In addition to its implications for functional recovery [5], moderate-intensity aerobic exercise is an integral component of lifestyle and risk factor management to prevent recurrent stroke and other major cardiovascular events [6]. Although recent evidence suggests an increased focus on incorporating aerobic exercise recommendations during formalized stroke rehabilitation [7], sedentary activities remain the dominant behavior of the inpatient environment [8,9,10,11,12]. Furthermore, stroke survivors are not meeting physical activity targets once discharged into the community [13,14]. The limited treatment time available to each patient, and a lack of access to the specialized equipment needed to adapt traditional ergometer-type aerobic exercise for stroke survivors with hemiparesis, are among the barriers to incorporating aerobic exercise during formalized rehabilitation [15]. Similarly, stroke related impairments limit access to existing exercise programs available at the community level [16]. Accordingly, practical solutions are needed to increase access to moderate-intensity aerobic exercise throughout the continuum of stroke recovery.

Task-oriented circuit training may be an effective strategy to replicate the intensities and outcomes achieved during traditional aerobic exercise training without using ergometers or other specialized equipment. A relatively large (N = 150) randomized control trial demonstrated that task-oriented circuit training was more effective than usual physiotherapy on gait outcomes in a cohort of stroke survivors who recently completed inpatient rehabilitation [17]. Other studies have reported beneficial effects of group-based exercise classes on cardiorespiratory fitness [18,19,20] and cardiometabolic risk factors [19] among community dwelling stroke survivors. However, the aerobic workloads maintained during such group-based exercise classes were not well defined. Furthermore, the extent to which task-oriented circuit training can achieve the large treatment effects reported for ergometer-type aerobic exercise interventions [21] needs further investigation.

Building on previous literature [17,18], we developed a task-oriented circuit training protocol that included activities typically employed during formalized stroke rehabilitation and did not require the use of ergometers or other specialized equipment. Task-oriented activities were incorporated into circuits that paired more metabolically demanding movements with less demanding ones to maintain a target heart rate range of 30 to 50 beats per minute above resting levels. Indeed, workloads supported during this intermittent functional training (IFT) protocol were sufficient to achieve at least moderate-intensity aerobic exercise criteria over a single session [22]. However, it is unknown whether such workloads are sustainable over a typical treatment period (i.e., 8 to 12 weeks) or if they can replicate the outcomes observed after ergometer-type aerobic exercise training. Accordingly, the current study aimed to evaluate participants’ ability to sustain moderate-intensity aerobic workloads during IFT over the 10-week study period and investigate its preliminary effects on cardiorespiratory fitness and metabolic profiles compared to ergometer-type aerobic exercise training.

## 2. Materials and Methods

### 2.1. Study Design

A parallel-groups randomized comparative study design was employed to evaluate participants’ ability to maintain moderate-intensity aerobic workloads during IFT and to investigate its preliminary effects compared to constant-load ergometer training (CET) performed on the treadmill according to best practice recommendations [23]. Participants who received inpatient and outpatient therapy from the provincial tertiary rehabilitation hospital within the preceding three years were recruited. The following inclusion criteria were used: (1) age ≥18 years, (2) ischemic or hemorrhagic stroke >6 months, (3) ability to perform two-step instruction, (4) and ambulatory with or without aid >10 m. Participants were excluded if they presented with any absolute contraindications for graded exercise testing as described elsewhere [24]. Allowing for a 20% dropout rate, we enrolled 20 participants per group to retain at least 16 participants in each exercise group. Power analysis based on previous studies employing similar dosages of aerobic exercise training [25,26] suggested that this sample size would have sufficient power to detect within-group differences in VO_2max_ over the study period (0.85 power, α = 0.05). Experimental procedures were approved by the regional Health Research Ethics Board (HREB# 2018.082), and all participants provided written informed consent following TCPS 2: Ethical Conduct for Research Involving Humans [27]. Participants were randomized in permuted blocks of 6-8 (using an opaque envelope) to receive either the IFT or CET interventions 3 days per week over 10 weeks. Given the nature of the study, it was not possible to blind participants on group assignments. Participant characteristics (age, sex, height, weight, and list of current medications), stroke history (date of onset, type of stroke), the severity of residual impairment using the National Institutes of Health Stroke Scale (NIHSS), and stage of lower limb recovery using the Chedoke McMaster scale [28], were recorded after randomization.

### 2.2. Interventions

Resting heart rate (HR) and blood pressure were recorded before each 30 min session of IFT and CET using a digital blood pressure monitor (Essentia, Physiologic, Montreal, Quebec). As described previously [22], IFT sessions involved performing 6–9 circuits of task-oriented exercises (3 different tasks per circuit) focused on improving functional ability. Circuits lasted approximately 3 min each, pairing more metabolically demanding tasks with less demanding ones in such a way as to maintain an HR range 30–50 beats per minute above resting levels. This target HR range is associated with at least moderate levels of aerobic intensity (i.e., >40% of heart rate reserve, HRR) in chronic stroke survivors [22] and reflects a 3 to 5 fold increase in HR compared to current inpatient rehabilitation practices [8]. Task-oriented exercises were selected based on each participant’s functional limitations, and difficulty was progressed throughout IFT (i.e., increasing the number of repetitions within a given time frame, increasing step height, incorporating unstable surfaces, etc.). Exercises included practicing rolling side to side on a mat, moving from lying to sitting and sitting to standing, stepping and transferring from standing position to the floor and back. Among participants randomized to CET, sessions were performed on a motorized treadmill (Trackmaster TMX58, Full Vision Inc., KS, USA) with harness support (<10% of body weight, PneuWeight, Pneumex, ID, USA) unless contraindicated and the total body recumbent stepper (T4r, Nustep, LLC, MI, USA) was used instead. Treadmill speed and incline (or load level and steps per min) were adjusted to achieve a target HR range corresponding to 40 to 60% of HRR as tolerated. The target HR range was calculated as follows:

40% HRR = (HRmax − HRrest) * 0.40 + HRrest(1)

60% HRR = (HRmax − HRrest) * 0.60 + HRrest(2)

where maximum HR (HRmax) was recorded during the baseline graded exercise test and resting HR (HRrest) was measured before each exercise session.

In terms of progression, the focus was on increasing treadmill workload (i.e., speed and incline). Continuous verbal encouragement was provided throughout both interventions to sustain workloads >40% of HRR. Exercise sessions were offered between 8:00 a.m. and 4:00 p.m. on Monday, Wednesday, and Friday each week over the intervention period. Exercise treatments were prescribed and monitored by two physiotherapists and a clinical exercise physiologist. Accessible transportation was available to all participants.

### 2.3. Outcome Measures

The primary outcome measure was the proportion of participations sustaining the prescribed moderate-intensity aerobic workloads (i.e., >40% of HRR) in 80% or more of the exercise sessions. Participants wore a chest strap HR transmitter (T31-Coded, Polar Electro Oy, Kempele, Finland) and a wrist worn wireless receiver (FT2, Polar Electro Oy, Kempele, Finland) during each session to monitor the HR response. The average HR was recorded for each 30 min IFT and CET session.

Secondary outcomes included comparing proportions of participants in the IFT and CET groups who achieved the a priori threshold for change in VO_2max_ and pre to post changes in parameters related to the metabolic profile. The threshold for meaningful change in VO_2max_ was set at >1.4 mL min^−1^ kg^−1^ based on the results of previous studies in chronic hemiparetic stroke survivors [25,26,29]. Using similar treadmill aerobic exercise training methods to the current research, Macko et al. (2005) reported a mean difference and 95% confidence interval for the difference between pre-and post-graded exercise tests of 2.1 mL min^−1^ kg^−1^ (95% CI: 1.4 to 2.7) over a 3 month period [26]. Other measures of cardiometabolic risk included changes in body mass index (BMI), resting blood pressure, resting HR, C-reactive protein (a measure of chronic inflammation), lipid profile (triglycerides, total cholesterol, high-density lipoprotein cholesterol (HDL), low-density lipoprotein cholesterol (LDL)), and short term glycemia (fructosamine). As an exploratory outcome, pre to post changes in whole-body resting energy expenditure was evaluated in a subset of the participants. Assessments of VO_2max_ and resting energy expenditure were performed over two sessions (at least 3 days apart) at baseline and after the intervention period. Participants were instructed to refrain from structured physical activities for 24 h and to consume no food for at least 4 h before each assessment. In an attempt to reduce biological variation, assessments were performed at the same time of day, and participants were instructed to take medications as prescribed throughout the trial.

#### 2.3.1. Maximum Oxygen Uptake

A calibrated indirect calorimetry system (Moxus, AEI Technologies, Pittsburgh, PA, USA) was used to assess VO_2max_ and resting whole-body energy metabolism. Symptom-limited graded exercise tests were performed on either the treadmill (with 10% body weight support) or the total body recumbent stepper to determine VO_2max_. The first two cohorts of participants in each group (n =14) performed pre-and post-graded exercise tests on the treadmill, and the remaining assessments were performed on the total body recumbent stepper. The decision to switch to a total body recumbent stepper was made due to challenges in determining the oxygen uptake/workload relationship on the treadmill. Although the bodyweight support harness was used and participants were instructed not to hold onto the handrail, participants felt unsteady with increasing workloads and repeatedly held onto the handrails. Participants were interfaced with the metabolic cart using 51 mm corrugated tubing (2.74 m) connected to a silicone oronasal face mask (8900 series) with a two-way non-rebreathing valve and headgear (Hans Rudolph Inc., Shawnee, KS, USA). Ventilatory parameters (tidal volume, breathing frequency, and minute ventilation) and expired gas concentrations (fraction of expired oxygen and fraction of expired carbon dioxide) were recorded breath by breath to determine rates of oxygen uptake (VO_2_) and carbon dioxide production (VCO_2_). The HR response to graded exercise testing was recorded in line with ventilatory parameters using a telemetry system (Polar Electro Oy Kempele, Finland) or electrocardiography device (CardioCard, Nasiff Associates Inc., Central Square, NY, USA), which were integrated with the indirect calorimetry system. Electrocardiography was only used when deemed necessary by the study physician. Rating of the perceived exertion was taken during the last 30 sec of each stage using the Borg CR10 scale [30,31]. The treadmill graded exercise test protocol was based on best practice recommendations [23], which involved walking at a self-selected speed and 0% incline for 2 min, followed by a 2.5% increase in grade every 2 min until a slope of 10% was reached and, after that, a 0.05 m/s increase in speed every 2 min, until test termination. The total body recumbent stepper protocol was adapted from previous work in this population [32] and involved increments in workload (~20 W) every 2 min through load level or step frequency changes. All testing was terminated according to established criteria [24]. Achievement of VO_2max_ was assessed based on the attainment of at least two of the following criteria [33]: (i) a plateau in VO_2_ (<2.1 mL min^−1^ kg^−1^) with increasing workload, (ii) respiratory exchange ratio greater than 1.15, and (iii) HRmax >90% of age-predicted maximum heart rate calculated as [34,35]:

HRmax = 207 − (0.7 * age)(3)

HRmax (beta-blockers) = 164 − (0.7 * age)(4)

Equation (4), was used to predict HRmax in participants receiving beta-adrenergic blockade therapy.

#### 2.3.2. Resting Energy Metabolism

To explore exercise-induced changes in whole-body energy metabolism, resting metabolic rate was added to the experimental protocol after the first two blocks of participants had completed the intervention period. Briefly, the protocol involved participants lying comfortably on an adjustable bed with their head supported by a single pillow in a temperature controlled (22–24 °C) and dimly lit room. Participants were instructed to minimize movements during the test and not to fall asleep. The indirect calorimetry system was adapted for resting metabolic rate measurements, which included the use of a flow-through canopy placed over the participant’s head and a flow meter (Moxus, AEI Technologies, Pittsburgh, PA, USA). According to manufacturer instructions, the flow rate was adjusted to maintain a fraction of expired oxygen between 0.7 and 1.0. The data collection period lasted 40 min; of which the first 15 min and last 5 min data were discarded. The remaining 20 min was averaged to determine VO_2_ and VCO_2_. Measurements were then used to calculate resting energy expenditure (REE), lipid oxidation rate (Lox), and carbohydrate oxidation rates (CHOox) according to the following formulas [36]:

REE = 3.91 VO_2_ + 1.10 VCO_2_ − 1.93 N (5)

Lox = 1.69 VO_2_ − 1.69 VCO_2_ − 2.03 N (6)

CHOox = 4.57 VCO_2_ − 3.23 VO_2_ − 2.60 N (7)

where urinary nitrogen excretion rate (N) was estimated to be 0.01 g min^-1^ [35]. Resting metabolic rate was also predicted (REEpred) using the Mifflin-St Jeor equations [37,38]:

REEpred (males) = 10 * weight + 6.25 * height − 5 * age + 5(8)

REEpred (females) = 10 * weight + 6.25 * height − 5 * age − 161(9)

where weight is measured in kg, height in cm, and age in years.

#### 2.3.3. Blood Analysis

Blood samples were taken immediately before the graded exercise tests and performed at baseline and post-intervention (3 days after the last exercise session). A 10 mL sample was obtained from the antecubital vein using (no additive) Vacutainer tubes (BD, Canada). The blood was left to clot at room temperature for 30 min, spun at 2200 rpm for 10 min, aliquoted into microcentrifuge tubes, and placed at −80 °C until blood chemistry tests were performed. Upon completing data collection, samples were thawed and analyzed at a local clinical laboratory using the methodologies described below. Briefly, CRP, total cholesterol, high-density lipoprotein cholesterol (HDL), triglycerides, and fructosamine were measured on Architect c16000 clinical chemistry systems (Abbott Diagnostics, Abbott Park, IL, USA). All testing reagents were purchased from Abbott Diagnostics. The CRP method was calibrated for high sensitivity CRP determination. The Friedwald Equation was used to calculate LDL cholesterol concentrations (LDL (mmol/L) = total cholesterol (mmol/L)—HDL (mmol/L)—(triglycerides (mmol/L)/2.2)) [39,40]. Fructosamine analysis included reference values between 205 and 285 µmol/L as normal for short-term glycemia [41]. The reference values for risk stratification based on CRP were as follows: <1 mg/L = low risk, 1–3 mg/L = moderate risk, and >3 mg/L = high risk of CVD [42]. The recommendations of the Expert Panel on Detection and Treatment of High Blood Cholesterol in [43] were used for risk assessments based on lipid profiles.

### 2.4. Statistical Analysis

Continuous data were first inspected for outliers, and distribution tested for normality using the D’Agostino–Pearson normality test. Differences between IFT and CET groups at baseline were evaluated using the independent samples t-test for continuous data, the Mann–Whitney U test for ordinal data, and Fisher’s exact test for nominal data. Differences in proportions of individuals maintaining at least moderate-intensity aerobic exercise workloads over the intervention period between IFT and CET were evaluated using Fisher’s exact test. Similarly, Fisher’s exact test was used to test for differences in proportions of individuals exceeding the 1.4 mL min^−1^ kg^−1^ threshold set for changes in VO_2max_ between the two groups. Within-group changes over the intervention period were evaluated using the paired samples t-test. The Wilcoxon matched-pairs test was used to evaluate blood marker changes due to the non-continuous nature of these data. Bland–Altman analysis was performed to assess the agreement between measured and predicted resting energy expenditure. Spearman correlation coefficients (rs) were used to evaluate associations between changes in VO_2max_ and changes in other metabolic markers. Statistical significance was set at *p* < 0.05. All statistical analyses and figures were performed in Prism 8 for MacOS (GraphPad Software, CA, USA).

## 3. Results

**Figure 1:**
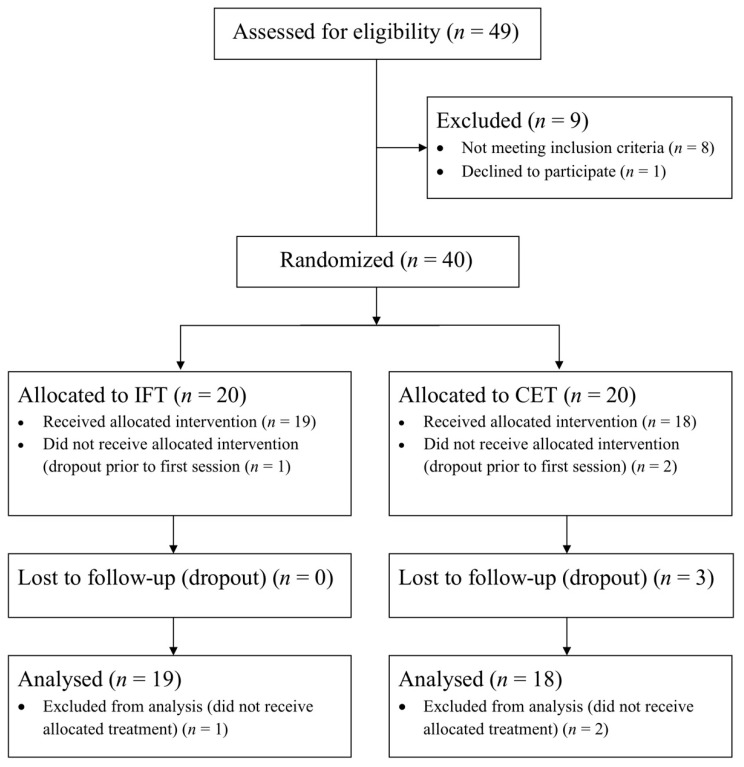
Participant flow through each stage of the study.

Of the 40 participants (65 years of age (SD 9)) randomized to receive either IFT or CET, 37 completed at least one session of their allocated treatment. As reported in Figure 1, no dropouts were observed among participants randomized to IFT, while 3 participant dropouts were observed in the CET group. Furthermore, two participants could not safely complete 30 min of walking on the treadmill and were switched to the total body recumbent stepper (participant numbers CET08 and CET15). As reported in Table 1, 35% of the randomized participants were female, and most suffered an ischemic stroke (31/40). No statistically significant group differences were observed for baseline characteristics. The average BMI was 27.8 kg m^−2^ (SD 4.7), corresponding to the overweight category based on the American College of Sports Medicine classification [24]. Although participants were, on average, evaluated 34 months (SD 29) after their first disabling stroke, many survivors were left with significant impairment. Accordingly, 63% of participants had NIHSS scores >5 or Chedoke-McMaster Stage of Recovery Leg or Foot scores <6, indicating moderate levels of impairment [28,44]. On average, participants had very poor levels of cardiorespiratory fitness 16.0 mL min^−1^ kg^−1^ (SD 5.0) according to age and sex-based normative data [24]. In fact, 60% had scores below the minimum threshold required for completing daily activities without inducing excessive fatigue (i.e., 18 to 20 mL min^−1^ kg^−1^) [25,45]. Accordingly, the current cohort represents the full range of impairments and functional limitations observed in community dwelling stroke survivors.

**Figure 2:**
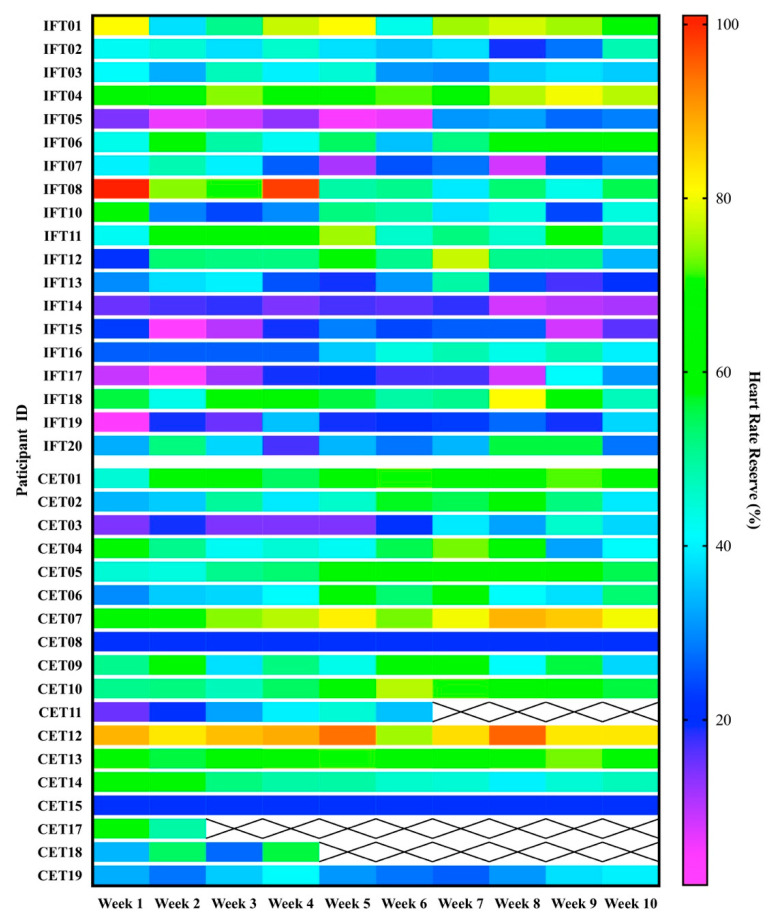
Dose analysis. Heatmap displaying participant’s average heart rate during each week of aerobic exercise training. The graph’s top and bottom halves are separated for intermittent functional training (IFT) and constant-load ergometer training groups (CET), respectively. Dark blue and purple cells indicate workloads below the moderate-intensity aerobic exercise threshold (i.e., <40% HRR).

Seven participants in the IFT and nine participants in the CET groups maintained >40% of HRR in at least 80% of the sessions (Figure 2). Statistical analysis (IFT *n* = 19, CET *n* = 18) revealed no differences between groups for proportions of participants meeting the moderate-intensity aerobic exercise criteria (Fisher’s exact test; *p* = 0.515). In addition, the impairment level did not appear to explain the relatively high proportions of participants not sustaining the prescribed workloads. For instance, of the participants who did not maintain >40% HRR, only 4/12 in the IFT group and 3/8 in the CET group had NIHSS >5 suggestive of moderate disability. Participants randomized to CET and switched to the total body recumbent stepper for their training completed 30/30 of their exercise sessions. However, they were unable to maintain workloads >40% of HRR.

**Figure 3:**
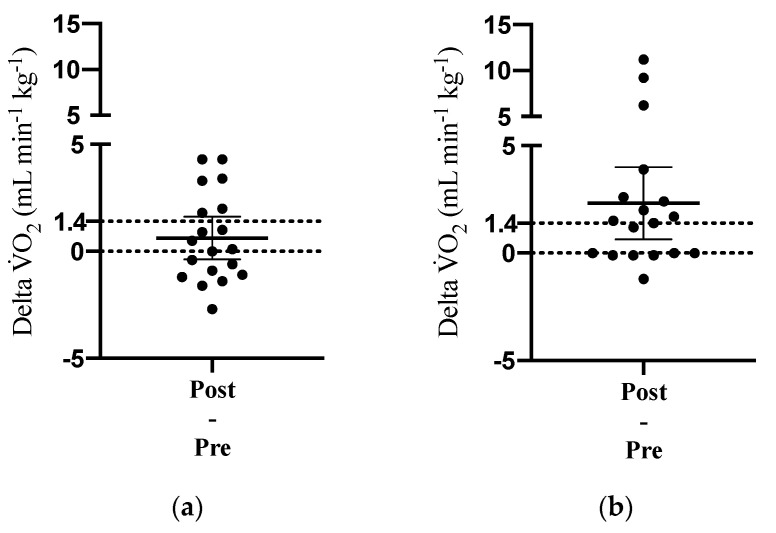
Change in maximum oxygen uptake (VO_2max_) over the 10-week intervention period among participants randomized to (**a**) intermittent functional training and (**b**) constant-load ergometer training. Dotted lines indicate the a priori threshold for change in VO_2max_ (i.e., 1.4 mL min^−1^ kg^−1^). Solid lines display the mean difference and 95% confidence interval.

Six participants in the IFT group and 11 participants in the CET group exceeded the 1.4 mL min^−1^ kg^−1^ threshold for change in VO_2max_. The difference in proportions between the two groups did not reach the level of statistical significance (Fisher’s exact test; *p* = 0.103). However, as displayed in Figure 3, CET was associated with more consistent and larger cardiorespiratory fitness changes. The mean difference and 95% confidence interval for the difference between pre and post measurements were 0.62 mL min^−1^ kg^−1^ (CI: −0.38 to 1.62) and 2.79 mL min^−1^ kg^−1^ (CI: 0.84 to 4.74) for participants randomized to IFT and CET, respectively. In terms of symptom-limited graded exercise testing to evaluate changes in cardiorespiratory fitness, no adverse events were observed, and most participants (n = 35) reached volitional exhaustion or were unable to maintain the target workload during the final stage. Two participants stopped the graded exercise test due to leg pain during baseline testing only. During the pre- and post-intervention assessments, 54 and 56% of participants reached at least two of the a priori criteria for determining VO_2max_, respectively (see Table 2).

Resting blood pressure and BMI measurements remained relatively stable over the study period across both groups (systolic (MD = −6 mmHg, CI: −14 to 2); diastolic (MD = 1 mmHg, CI: −5 to 6); BMI (MD = −0.20 kg m^−2^, CI: −0.43 to 0.03)). However, a consistent decrease in pre to post assessments of resting heart rate was observed after IFT (MD = −4.1 bpm, CI: −7.4 to −0.7) and CET (MD = −5.9 bpm, CI: −10.0 to −1.8). Average serum CRP exceeded the high risk reference values at both time points among participants randomized to IFT (Pre: 4.3 mg/L (SD 5.1); Post: 3.7 mg/L (SD 4.2) and CET (Pre: 5.3 mg/L (SD 6.1); Post: 4.0 mg/L (SD 5.7)) However, four participants in each group dropped their scores into a lower risk category, which was associated with statically significant pre to post changes in CRP across both groups (MD = −0.9 mg/L CI: −1.9 to −0.1). As displayed in Table 3, the majority of participants had serum fructosamine levels within the normal range (200 to 285 µmol/L) at both time points. However, average scores increased after IFT (MD = 11.5 µmol/L, CI: −12.7 to 35.8) and CET (MD = 18.7 µmol/L, CI: 3.2 to 34.1), which suggests an increase in short-term glycemia after the exercise interventions. Similarly, scores for total cholesterol, HDL, LDL, and triglycerides were associated with low risk at baseline and post intervention for both exercise groups. A small but statistically significant increase in total cholesterol was observed after IFT (MD = 0.2 mmol/L, CI: 0.04 to 0.39). In terms of correlations between changes in VO_2max_ and metabolic markers, the only statistically significant association observed was with changes in fructosamine (rs = 0.42, *p* = 0.024).

**Figure 4:**
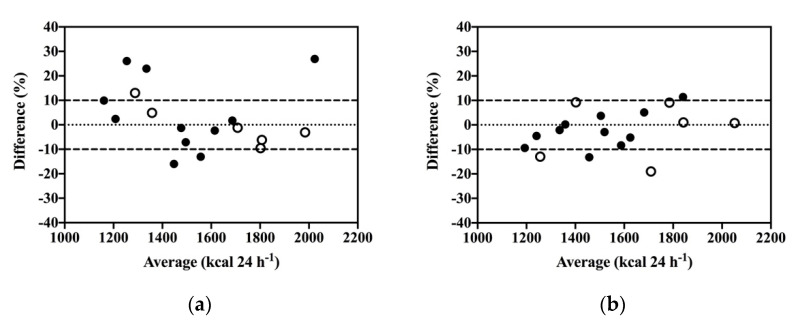
Bland–Altman analysis of agreement between measured resting energy expenditure via indirect calorimetry and predicted energy expenditure via the Mifflin-St Jeor equation during pre (**a**) and post assessments (**b**). IFT (*n* = 11) closed circles; CET (*n* = 6) open circles.

**Figure 5:**
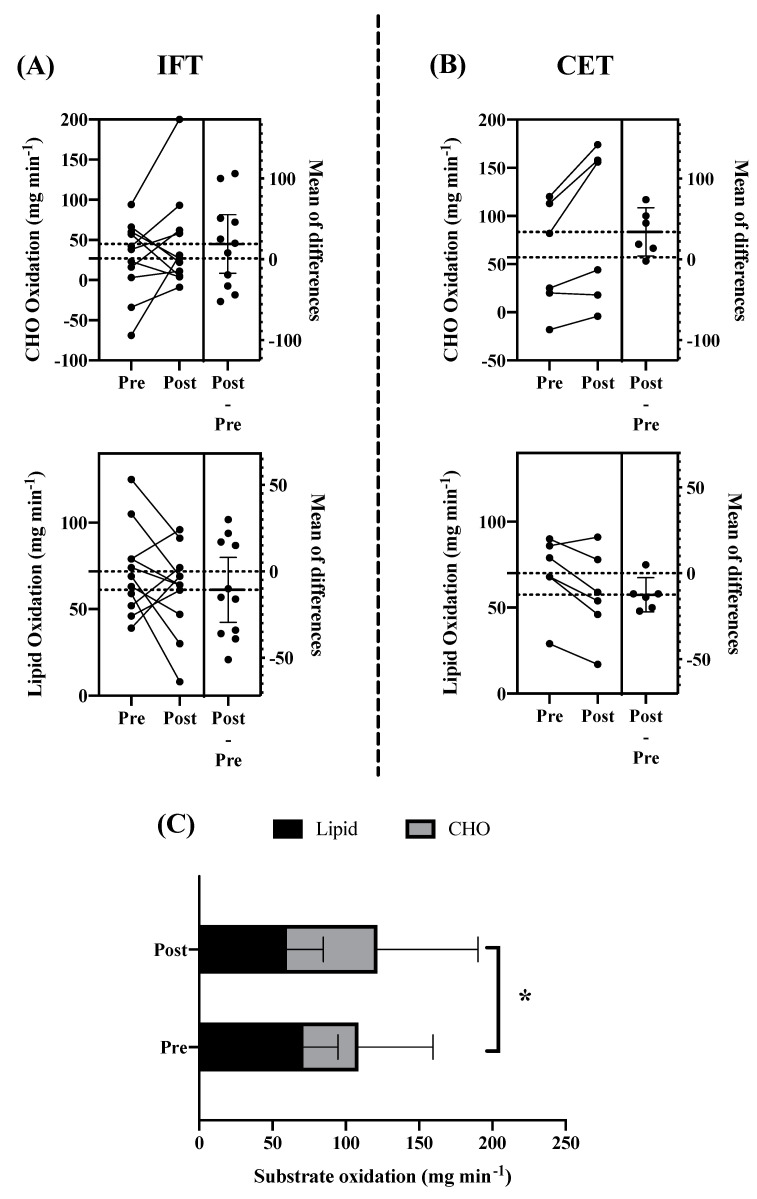
Resting Substrate Partitioning. Changes in rates of resting carbohydrate (CHO) and lipid oxidation over the intervention period. (**A**) Intermittent functional training (IFT), (**B**) constant-load ergometer training (CET), and (**C**) combined groups. Panels A and B display individual changes along with mean difference and 95% CI. Panel C displays pre to post changes in substrate partitioning across both groups, * *p* < 0.05.

The resting indirect calorimetry data indicated a shift in whole-body energy metabolism over the study period. Although little change in pre to post measurements of daily resting energy expenditure was observed (MD = −21 kcal 24 hr^−1^, CI: −103 to 61), the agreement between measured and predicted values was improved after exercise training compared to baseline. Bland–Altman analysis of the percent difference between measured and predicted resting energy expenditure (100 * (measured – predicted)/average) revealed the bias and 95% confidence interval of the agreement to be 2.82 (CI: −22.72 to 28.38) and −2.19 (−19.02 to 14.64) at pre and post assessments, respectively. As displayed in Figure 4, the discrepancy between measured and predicted values decreased from pre to post measurements. This change was coupled with a shift in resting substrate partitioning toward increased reliance on carbohydrate energy substrates. As displayed in Figure 5, lipid oxidation rates decreased, and carbohydrate oxidation rates increased from pre to post resting metabolic rate measurements after IFT (Lipid: MD = −10.6 mg min^−1^ CI: −29.4 to 8.1; CHO: MD = 18.8 mg min^−1^ CI: −17.4 to 55.2) and CET (Lipid: MD = −12.5 mg min^−1^ CI: −22.5 to −2.5; CHO: MD = 34.0 mg min^−1^ CI: 4.2 to 63.8).

## 4. Discussion

We undertook this study to evaluate chronic hemiparetic stroke survivors’ ability to sustain moderate-intensity aerobic workloads during IFT over the 10-week study period and to investigate its preliminary effects on cardiorespiratory fitness and metabolic profiles. Compared to best practice recommendations for ergometer-type aerobic exercise training, a similar proportion of participants randomized to IFT maintained the moderate-intensity aerobic exercise criteria over the 10-week study period. Accordingly, organizing task-oriented activities into 3 min circuits that paired more metabolically demanding tasks with less demanding ones to increase HR 30 to 50 beats per minute above resting levels was a practical method to replicate the intensities of aerobic exercise imposed during ergometer-type training. The added value of such aerobic exercise strategies includes the fact that it does not require the use of specialized equipment, and task-oriented activities can be adapted to individuals’ levels of impairment, making IFT a practical tool to provide moderate-intensity aerobic exercise throughout the continuum of stroke recovery.

Previous studies have evaluated the effects of different functional exercise training paradigms among individuals recovering from a stroke [17,18,20,46]. However, the aerobic workloads maintained during prior investigations were not well defined, and to date, no comparison has been made with traditional ergometer-type aerobic exercise training. Among the ergometers appropriate for hemiparetic stroke survivors, the bodyweight supported treadmill is perhaps the best choice for comparison with functional exercise because it incorporates both task practice and aerobic stress [47,48]. Also, several randomized control trials confirmed the beneficial effects of treadmill aerobic exercise training on cardiorespiratory fitness and metabolic outcomes among stroke survivors during the subacute [47,48] and more chronic phases of recovery [25,26,29,49]. However, in the current study, almost half of the participants randomized to CET failed to sustain the prescribed aerobic workloads. The extent to which this observation aligns with previous interventional studies is unclear because previous authors often neglected to report the actual aerobic workloads maintained throughout the study period. However, it does align with previous feasibility studies that have identified stroke survivors’ limited ability to sustain moderate-intensity workloads as barriers to implementing aerobic exercise guidelines during formalized rehabilitation [50]. Also, the within-group changes in cardiorespiratory fitness observed after CET are similar to those previously reported [25,26,29]. Therefore, the dose analysis likely reflects a true variability among chronic hemiparetic stroke survivors in their ability or willingness to sustain moderate-intensity aerobic workloads.

Although some participants (n = 6) randomized to IFT exceeded the a priori threshold for anticipated change in VO_2max_, CET was associated with more extensive effects on cardiorespiratory fitness. The blunted effect of IFT likely reflects a lower total energy expenditure of exercise compared to CET. Given that both interventions were matched for treatment time, the rest periods incorporated during the intermittent protocol and the time taken to switch between task-oriented activities, reduced the total amount of time engaged in moderate-intensity aerobic exercise during IFT. Our previous cross-sectional study demonstrated that about 75% of the IFT session was categorized as exercise time [22]. Although the aerobic workloads achieved during IFT were substantially higher than those observed during task-oriented therapies performed during contemporary stroke rehabilitation [2,8], they were not high enough to overcome the decreased energy demand during rest periods. Therefore, IFT session duration will likely need to be increased to match the energy expended during CET before a valid comparison between interventions can be made. In contrast, high-intensity interval training protocols have been used to achieve shorter exercise durations while matching the energy expenditure of exercise [51]. The difference here is that high-intensity interval training interventions incorporate much higher relative workloads (e.g., 3 min at workloads >80% of VO_2max_) along with recovery intervals (e.g., 3 min at workloads <60% of VO_2max_) to match the energy expenditure of exercise with constant workload activities. The extent to which task-oriented activities can be used to achieve high-intensity aerobic intervals in hemiparetic stroke survivors is beyond the current study’s scope. However, the present data demonstrate that the moderate-intensity aerobic workloads sustained during IFT are sufficient to increase cardiorespiratory fitness for at least some stroke survivors.

Serum CRP was the most responsive blood marker to change in the current study. Elevated CRP levels are associated with an increased risk of future cardiovascular events [52] and unfavorable long-term functional outcomes after ischemic stroke [53]. Four participants in each group reduced their CRP levels into a lower risk category, and statistically, significant pre to post changes were observed across both groups. The observed changes in CRP align with the results of previous exercise interventions [54] and highlight the value of including this biomarker of cardiovascular risk in future definitive trials among stroke survivors.

Short-term glycemia and lipid profiles remained within normal and low-risk reference values throughout the study period. However, statistically significant within-group increases in serum fructosamine and total cholesterol were observed after CET and IFT, respectively. The observed changes are in contrast with a recent meta-analysis on the topic, which reported decreases in fasting glucose and total cholesterol [55]. Similarly, the observed increase in carbohydrate oxidation during post-intervention assessments of resting metabolic rate is inconsistent with the concept of improved metabolic fitness [56]. Given that dietary controls were not imposed in the current study, the increase in short-term glycemia and the shift toward increased oxidation of carbohydrate energy sources at rest could result from changes in eating behaviors over the study period. However, the ability to switch between energy substrates is depressed among deconditioned populations [57,58], and the observed changes in substrate partitioning might reflect a positive acclimation to exercise training in this cohort. In fact, a small but statistically significant positive association was observed between changes in VO_2max_ and serum fructosamine levels. Also, the agreement between predicted and observed resting energy expenditure improved over the study period. More studies are needed to elucidate the dose–response effects of exercise on metabolic flexibility in clinical populations such as stroke survivors.

Among the limitations of the current study was that almost half of the study participants failed to achieve the a priori criteria for valid VO_2max_ measurement. This is a common observation in stroke rehabilitation trials, and because of this, many research groups describe the results of graded exercise testing as “peak oxygen uptake” rather than maximum [26,47,59,60,61]. However, without a valid assessment of VO_2max_, it is difficult to interpret the pre to post changes in graded exercise testing [62]. Therefore, new exercise testing protocols are needed to improve the validity of VO_2max_ measurements in clinical populations such as stroke survivors. As described above, the lack of dietary controls may have influenced outcomes, and future investigations should monitor changes in eating and physical activity behaviors over the study period. Finally, the current study was not powered to determine equivalence between the two interventions. Although the difference in proportions of participants achieving the threshold for the anticipated change in VO_2max_ did not reach the level of statistical significance, these data should be interpreted with caution.

## 5. Conclusions

Task-oriented circuit training can replicate the aerobic intensities sustained during traditional ergometer-type exercise training among chronic hemiparetic stroke survivors. However, relatively large proportions of participants in both exercise groups failed to achieve the moderate-intensity aerobic exercise criteria over the 10-week study period. Accordingly, more studies are needed to elucidate the individual variability in sustaining moderate-intensity aerobic workloads among participants with similar levels of impairment. Also, more work is needed to evaluate the extent to which task-oriented circuit training can be used to replicate the outcome observed after ergometer-type aerobic exercise.

## Figures and Tables

**Table 1:** Baseline Characteristics.

ID	Sex	Age	BMI (kg m^−2^)	Stroke Type	NIHSS	Chedoke (Leg/7)	Chedoke (Foot/7)	VO_2max_ (mL min^−1^ kg^−1^)
IFT01	F	58	21.5	Hemorrhagic	2	5	4	22.9
IFT02	M	61	38.9	Ischemic	1	6	7	22.8
IFT03	M	50	27.6	Hemorrhagic	17	3	2	14.6
IFT04	M	75	24.5	Ischemic	3	6	5	23.5
IFT05	M	66	32.1	Ischemic	15	3	1	11.8
IFT06	M	79	28.4	Hemorrhagic	11	3	2	15.9
IFT07	F	79	26.2	Ischemic	0	6	6	14.2
IFT08	M	67	29.6	Ischemic	3	6	6	19.6
IFT09	M	69	28.5	Ischemic	1	-	-	15.8
IFT10	M	81	28.2	Hemorrhagic	3	6	6	9.9
IFT11	M	74	28.0	Hemorrhagic	7	5	5	17.2
IFT12	M	79	29.5	Ischemic	9	3	2	10.8
IFT13	M	54	26.8	Ischemic	9	5	3	14.3
IFT14	M	71	28.4	Hemorrhagic	10	5	4	12.0
IFT15	F	73	24.7	Ischemic	3	5	6	8.0
IFT16	F	63	26.7	Ischemic	2	5	5	19.3
IFT17	F	70	27.9	Ischemic	1	7	7	13.5
IFT18	M	55	32.9	Ischemic	3	7	7	20.2
IFT19	F	64	18.3	Hemorrhagic	0	7	7	19.7
IFT20	M	71	32.9	Ischemic	3	7	7	19.6
CET01	M	71	30.9	Ischemic	6	5	6	15.1
CET02	M	43	24.3	Ischemic	7	3	1	17.6
CET03	M	62	26.9	Ischemic	4	5	2	19.3
CET04	F	49	29.6	Hemorrhagic	5	5	4	14.5
CET05	M	69	26.3	Ischemic	1	7	6	20.0
CET06	M	67	24.2	Ischemic	12	3	4	15.2
CET07	M	69	19.1	Ischemic	6	5	4	19.5
CET08	F	71	35.3	Ischemic	15	1	1	4.9
CET09	M	65	35.6	Ischemic	0	7	7	15.1
CET10	M	52	32.9	Ischemic	1	6	7	23.1
CET11	F	72	24.3	Hemorrhagic	3	5	5	-
CET12	F	72	22.8	Ischemic	5	5	4	10.2
CET13	M	54	29.6	Ischemic	10	3	1	18.4
CET14	M	58	37.8	Ischemic	3	6	5	10.8
CET15	M	78	22.7	Ischemic	9	5	3	7.9
CET16	F	67	21.1	Ischemic	-	-	-	11.8
CET17	M	60	24.1	Ischemic	2	-	-	28.3
CET18	F	52	30.6	Ischemic	0	6	5	18.2
CET19	F	66	26.0	Ischemic	1	6	6	12.0

**Table 2:** Participants Maximum Absolute Oxygen Uptake and Achievement of Graded Exercise Testing Criteria at Pre and Post Assessments.

ID	Ergometer	Pre-Graded Exercise Test					Post-Graded Exercise Test				
		VO_2_	Plateau Yes/No	RER	HRM	RPE (/10)	VO_2_	Plateau Yes/No	RER	HRM	RPE (/10)
IFT01	Treadmill	1467	yes	1.06	70	4	1716	No	1.06	82	5
IFT02	Treadmill	2878	yes	1.01	98	7	2924	Yes	1.00	93	7
IFT03	Treadmill	1458	yes	0.99	78	10	1540	Yes	1.07	79	10
IFT04	Treadmill	1782	yes	1.05	98	7	1716	No	1.17	82	3
IFT05	Treadmill	1131	yes	0.85	74	8	1331	Yes	0.89	75	10
IFT06	Treadmill	1272	yes	1.03	124	-	1246	Yes	1.09	124	-
IFT07	NuStep	865	yes	0.84	77	5	860	Yes	0.90	88	3
IFT08	NuStep	1776	no	1.12	98	7	1664	No	1.12	94	10
IFT09	NuStep	1348	yes	1.03	104	8	Dropout prior to intervention				
IFT10	NuStep	810	yes	0.97	96	7	1088	Yes	1.02	91	9
IFT11	NuStep	1526	no	1.09	120	5	1481	Yes	1.06	121	9
IFT12	NuStep	1013	yes	0.98	88	9	1017	Yes	1.01	84	5
IFT13	NuStep	1096	yes	1.06	83	5	1162	No	1.08	79	9
IFT14	NuStep	1000	yes	0.99	60	10	1339	No	1.05	67	7
IFT15	NuStep	548	yes	0.92	54	5	605	Yes	0.92	53	8
IFT16	NuStep	1189	no	1.07	94	10	1010	No	1.04	89	5
IFT17	NuStep	950	yes	1.04	74	9	920	Yes	1.05	66	10
IFT18	NuStep	1930	no	1.12	101	7	1745	No	1.07	100	5
IFT19	NuStep	1035	no	1.07	103	10	1260	Yes	1.07	101	10
IFT20	NuStep	1809	no	1.22	88	10	1727	No	1.23	87	7
CET01	Treadmill	1391	no	1.05	94	6	1250	Yes	1.10	93	5
CET02	Treadmill	1270	yes	0.96	77	8	1845	No	1.04	103	5
CET03	Treadmill	1676	no	0.94	80	6	2431	Yes	1.06	106	6
CET04	Treadmill	1204	yes	1.01	98	5	1290	Yes	1.01	98	5
CET05	Treadmill	1702	no	0.99	103	7	2235	Yes	1.07	104	2
CET06	Treadmill	1089	no	0.92	75	5	1217	Yes	0.97	96	-
CET07	Treadmill	1126	yes	1.21	91	10	1253	Yes	1.26	98	5
CET08	NuStep	397	yes	0.80	60	3	524	Yes	0.87	55	-
CET09	Treadmill	1804	yes	0.94	93	10	1836	No	1.05	93	10
CET10	NuStep	2350	no	0.97	90	10	2685	No	1.02	95	7
CET11	NuStep	1370	yes	1.00	83	7	Dropout during intervention				
CET12	NuStep	589	yes	1.07	77	5	588	Yes	1.16	70	4
CET13	NuStep	1647	no	1.17	84	10	1755	No	1.16	87	10
CET14	NuStep	1193	yes	1.07	76	7	1597	No	0.94	79	9
CET15	NuStep	587	yes	1.05	88	4	580	Yes	1.14	74	5
CET16	NuStep	680	yes	0.83	67	7	Dropout prior to intervention				
CET17	NuStep	2146	yes	1.05	84	7	Dropout during intervention				
CET18	NuStep	1591	yes	1.09	88	9	Dropout during intervention				
CET19	NuStep	818	no	1.12	98	7	976	Yes	1.09	93	7

**Table 3:** Pre to Post Changes in short-term glycemia and lipid profiles during intermittent functional training and constant workload ergometer exercise.

ID	Fructosamine (µmol/L)		Total Cholesterol (mmol/L)		High-Density Lipoprotein (mmol/L)		Low-Density Lipoprotein (mmol/L)		Triglycerides(mmol/L)	
	Pre	Post	Pre	Post	Pre	Post	Pre	Post	Pre	Post
IFT01	244.4	261.0	5.017	5.475	1.322	1.203	3.195	3.710	1.101	1.237
IFT03	279.6	281.7	3.606	3.782	1.137	1.110	1.889	2.026	1.275	1.422
IFT04	238.2	261	3.094	3.609	1.040	1.184	1.758	2.119	0.652	0.674
IFT05	186.4	198.9	3.106	3.154	0.991	1.038	1.665	1.600	0.989	1.136
IFT07	232.0	246.5	3.221	3.135	0.906	0.905	1.339	1.428	2.147	1.764
IFT08	294.1	298.3	2.743	2.755	0.862	0.862	1.234	1.192	1.424	1.543
IFT10	236.1	221.6	2.283	2.254	0.792	0.849	0.943	0.983	1.206	0.928
IFT11	258.9	244.4	3.116	3.363	1.471	1.316	1.239	1.587	0.893	1.013
IFT12	269.3	244.4	3.296	3.604	0.883	0.862	1.942	2.108	1.036	1.395
IFT14	366.6	372.9	4.924	6.032	1.407	1.824	2.717	3.102	1.759	2.433
IFT15	261	275.5	3.376	3.669	1.047	1.156	1.838	1.998	1.08	1.132
IFT16	209.2	209.2	4.215	4.220	1.551	1.565	1.806	2.052	1.887	1.326
IFT17	263.1	248.6	2.688	3.082	1.312	1.410	1.059	1.277	0.698	0.869
IFT18	256.9	242.4	2.329	2.710	0.656	0.739	1.049	1.443	1.373	1.162
IFT19	219.6	393.6	4.946	4.619	1.666	1.654	2.897	2.509	0.842	1.004
IFT20	279.6	279.6	3.572	3.501	1.043	1.054	1.768	1.571	1.675	1.927
CET01	194.7	190.6	2.774	2.75	0.926	0.844	1.223	1.314	1.375	1.303
CET02	354.2	426.7	3.532	3.114	0.956	0.854	1.998	1.756	1.271	1.109
CET03	225.8	254.8	5.53	5.326	1.111	1.125	3.809	3.541	1.343	1.453
CET04	203.0	203.0	5.261	6.105	1.322	1.541	3.068	3.883	1.917	1.498
CET05	225.8	236.1	2.534	2.498	1.107	1.115	0.835	0.768	1.302	1.352
CET06	263.1	292.1	2.933	3.303	0.731	0.954	1.736	1.917	1.025	0.95
CET07	265.1	285.9	2.098	2.263	0.801	0.925	1.012	0.959	0.627	0.833
CET09	171.9	192.6	4.066	3.483	1.134	1.107	2.409	1.926	1.151	0.99
CET10	209.2	219.6	3.407	3.811	1.666	1.931	1.371	1.558	0.813	0.709
CET12	265.1	263.1	3.544	3.135	1.369	1.222	1.606	1.336	1.251	1.27
CET13	341.8	401.9	3.383	3.263	1.238	1.104	1.386	1.584	1.67	1.266
CET14	298.3	277.6	3.917	3.729	1.082	0.995	2.408	2.279	0.939	1.001
CET19	169.9	186.4	2.647	2.565	0.971	0.994	0.946	0.776	1.606	1.749

## Data Availability

Data are available on reasonable request from the corresponding authors at the Memorial University of Newfoundland, Canada.
